# Variability in the Spatial Structure of the Central Loop in Cobra Cytotoxins Revealed by X-ray Analysis and Molecular Modeling

**DOI:** 10.3390/toxins14020149

**Published:** 2022-02-18

**Authors:** Peter V. Dubovskii, Kira M. Dubova, Gleb Bourenkov, Vladislav G. Starkov, Anastasia G. Konshina, Roman G. Efremov, Yuri N. Utkin, Valeriya R. Samygina

**Affiliations:** 1 Shemyakin-Ovchinnikov Institute of Bioorganic Chemistry, Russian Academy of Sciences, 16/10 Miklukho-Maklaya str., 117997 Moscow, Russia; vladislavstarkov@mail.ru (V.G.S.); konnij@gmail.com (A.G.K.); efremov@nmr.ru (R.G.E.); utkin@ibch.ru (Y.N.U.); 2 FSRC “Crystallography and Photonics”, Russian Academy of Sciences, 111933 Moscow, Russia; kira-d91@mail.ru (K.M.D.); lera@crys.ras.ru (V.R.S.); 3 NRC “Kurchatov Institute”, 123182 Moscow, Russia; 4 European Molecular Biology Laboratory, Hamburg Unit, c/o DESY, 22607 Hamburg, Germany; gleb@embl-hamburg.de; 5 Moscow Institute of Physics and Technology (State University), 9 Institutskiy per., 141700 Dolgoprudny, Russia; 6 Higher School of Economics, National Research University, 20 Myasnitskaya str., 101000 Moscow, Russia

**Keywords:** cobra cytotoxin, *Naja* *naja* venom, X-ray crystallography, cis–trans isomerism of X-Pro peptide bond, molecular dynamics, Highly Mimetic Membrane Model

## Abstract

Cobra cytotoxins (CTs) belong to the three-fingered protein family and possess membrane activity. Here, we studied cytotoxin 13 from *Naja naja* cobra venom (CT13Nn). For the first time, a spatial model of CT13Nn with both “water” and “membrane” conformations of the central loop (loop-2) were determined by X-ray crystallography. The “water” conformation of the loop was frequently observed. It was similar to the structure of loop-2 of numerous CTs, determined by either NMR spectroscopy in aqueous solution, or the X-ray method. The “membrane” conformation is rare one and, to date has only been observed by NMR for a single cytotoxin 1 from *N. oxiana* (CT1No) in detergent micelle. Both CT13Nn and CT1No are S-type CTs. Membrane-binding of these CTs probably involves an additional step—the conformational transformation of the loop-2. To confirm this suggestion, we conducted molecular dynamics simulations of both CT1No and CT13Nn in the Highly Mimetic Membrane Model of palmitoiloleoylphosphatidylglycerol, starting with their “water” NMR models. We found that the both toxins transform their “water” conformation of loop-2 into the “membrane” one during the insertion process. This supports the hypothesis that the S-type CTs, unlike their P-type counterparts, require conformational adaptation of loop-2 during interaction with lipid membranes.

## 1. Introduction

Three-finger toxins (TFTs) are disulfide-rich proteins from the venoms of cobras [1,2,3,4], kraits [5], coral [6], and some other snakes. Their fold features three distinct β-structural “fingers”, emerging from a globular core stapled by four conserved disulfide bridges [7,8,9,10]. These proteins target a wide variety of receptors, channels, and enzymes, serving as a source of valuable pharmacological tools [11,12,13,14,15,16].

Cardiotoxins, or cytotoxins (CTs), are one of the largest groups of TFTs, affecting the integrity of lipid membranes, and/or influencing the activity of membrane proteins and the associated signaling cascades [17,18]. Membrane activity of CTs is associated with the anticancer properties of these molecules [19]. Based on studies of the interactions of CTs with sphingomyelin vesicles and detergent micelles, these toxins were classified into P- and S-types [20]. The former feature the presence of Pro30, while the latter Ser28 residue. The P-type CTs exhibit minor structural changes upon their transfer from aqueous phase to the membrane environment [21]. The S-type ones demonstrate reorganization near Ser28 residue [22]. This can be observed by NMR spectroscopy via comparison of the spatial structure of CTs in aqueous solution and in the presence of detergent micelles. To date, only three CTs have been studied in detergent micelles [21,22,23]. However, from more than 80 known CTs [24], spatial models in this environment have been obtained for only two CTs, cytotoxins 1 and 2 from *N. oxiana* [21,22]. The effects of *N. oxiana* CTs on multilamellar vesicles of phosphatidylglycerol have been studied with ^31^P-NMR spectroscopy [25,26,27]. However, molecular details of these interactions are still absent. They can be elucidated in silico, but only few attempts, involving both coarse-grained (CG) [28] and all-atom (AA) [24,29,30,31] molecular dynamics (MD) simulations of CTs in membranes have been performed. CG-MD study of a CT in the POPC bilayer showed the importance of the structural changes near the Ser28-fragment for embedding of the toxin molecule into the membrane [22]. These structural changes, or adaptations to the membrane environment agree well with NMR data obtained for detergent micelles [21,22].

Due to the fact that CTs are one of the most abundant protein fractions in cobra venom, the latter remains the main source of CTs for various purposes. Only recently, recombinant production of these molecules has became a valuable alternative [32]. Solid-phase chemical synthesis of full-length CTs is far less popular due to low yields [33] and difficulties in correct closure up of disulfide bridges [34]. Several CT isoforms are present within the venom of any cobra species (e.g., [35]). For example, in *N. kaouthia* venom, several cytotoxins were identified, including close homologues such as cytotoxin 2 (CT2Nk) and cytotoxin 3 (CT3Nk) [36] (Table 1). Using NMR spectroscopy, we determined the spatial structure of these toxins without their separation [37]. Later, we found that the toxin, identical to CT2Nk is present in *N. naja* venom (marked as CT13Nn in Table 1).

Here, we determined the spatial organization of CT13Nn (CT2Nk, or L48V49 variant of CT3Nn *N.naja*) toxin by X-ray crystallography. We then studied the interaction of this toxin with the anionic membranes of palmitoiloleoylphosphatidylglycerol (POPG), using the Highly Mimetic Membrane Model (HMMM) [38]. Previously, this membrane model had not been used for studies of CT/lipid interactions. Its main feature is the presence of an organic solvent layer representing the hydrophobic core of the membrane, while short-tailed phospholipids constitute the headgroup region. These lipid molecules exhibit up to two orders of magnitude enhancement in lateral diffusion, leaving the membrane atomic density profile of the headgroup region essentially identical to that of the membrane models composed of full-length lipid molecules. Use of the HMMM looks promising because substantial acceleration (from microseconds to few hundred nanoseconds) compared to conventional all-atom MD simulation study of CTs can be achieved. In addition, we performed similar calculations for cytotoxin 1 from *N. oxiana* (CT1No) (Table 1). For this toxin, the spatial structure in aqueous solution and in dodecylphosphocholine (DPC) micelles had been determined earlier [22]. Thus, we were able to demonstrate that the spatial organization of the central loop in a CT molecule in the crystal state can be similar to the one adopted by this molecule in the lipid membrane. This information is important for establishing structure–activity relationships in CTs.

## 2. Results

### 2.1. X-ray Crystallography

**Figure 1:**
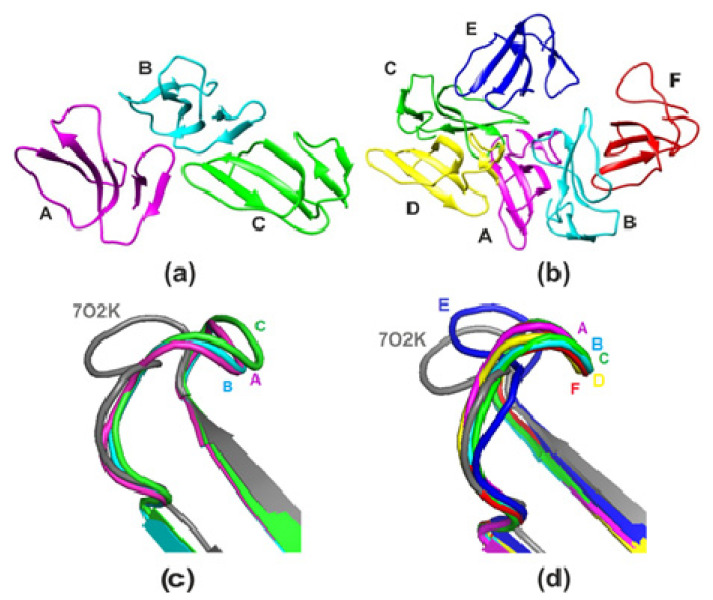
Structure of the hexagonal (**a**) and orthorhombic (**b**) crystal forms of CT13Nn. The subunitts are denoted A–F. Comparison of CT structures solved by NMR (CT2Nk in aqueous solution, model 7O2K) and X-ray (CT13Nn of the hexagonal (**c**) and orthorhombic form (**d**)). Protein is shown in cartoon representation. 7O2K is colored brown; the color scheme of subunits of the hexagonal and orthorhombic forms is the same in all panels.

Here, we used X-ray crystallography to determine the spatial organization of CT13Nn. Structures of hexagonal (with three molecules in the asymmetric unit denoted A, B, and C; Figure 1a) and orthorhombic (with six molecules in an asymmetric unit denoted A, B, C, D, E, and F; Figure 1b) crystal forms were solved at 2.3 and 2.6 Å-resolution, respectively.

It was impossible to accurately determine the amino acid sequence of the 47–50 region solely by mass spectrometry. Analysis of the 2Fo-Fc electron density map of the 2.3Å structure revealed that the correct variant is the LLVK sequence (Figure S1).

**Figure 2:**
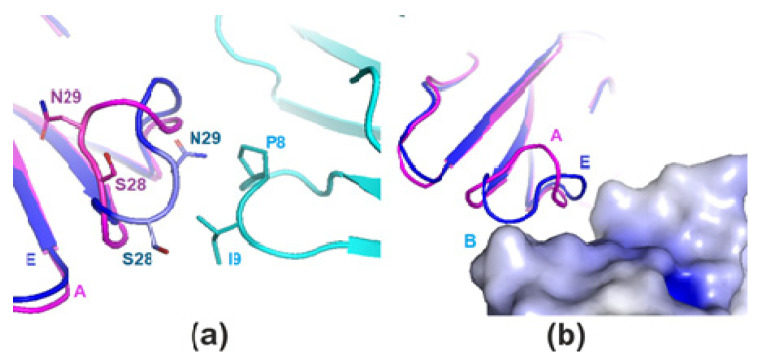
Influence of crystal packing on the conformation of loop-2 as exemplified with subunit A of the orthorhombic crystal form. (**a**) Subunits E (blue) and A (pink) are superimposed. Neighboring subunit B is colored cyan. Residues involved in repulsion in subunits E and B are labeled and given in stick representation. (**b**) Subunits A and E are shown in cartoon representation; electrostatic surface is shown for subunit B (areas with positive and neutral values of electrostatic potential are colored blue and grey, respectively).

The average value of the B-factor of protein atoms amounts to 38.9 Å^2^ and 98.5 Å^2^ in the hexagonal and the orthorhombic form, respectively. This indicates that the hexagonal form is more ordered. The molecules in the asymmetric part of each structure feature different level of disorder. The electron density for molecules D, E, and F of the orthorhombic structure is considerably poorer than for three other molecules, which is also reflected in the higher B-factor (Table S1). The most ordered molecules in the hexagonal and the orthorhombic structure are B and A, respectively. Subunit B of the hexagonal form was further used for conformational analysis and comparison with other NMR or X-ray structures. A root-mean-square deviation (RMSD) between coordinates of Cα atoms of molecule B in the hexagonal crystal form superimposed on the other two molecules ranges from 0.26 to 0.34 Å. RMSD for Cα atoms of molecule A of the orthorhombic structure superimposed on the other five molecules ranges from 0.39 to 1.45 Å. Significant difference between E subunit of the orthorhombic structure and most other subunits could be explained by an unusual conformation of loop-2 in all subunits, except E. Previously, this was not observed in crystal structures of homologues. This conformation was also found in all subunits of the hexagonal structure (Figure 2a).

**Figure 3:**
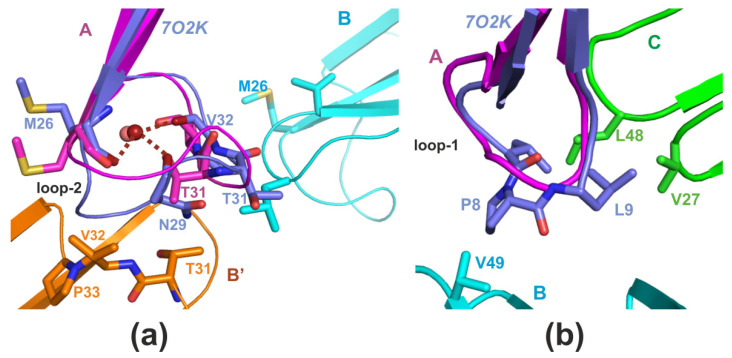
Superimposed loop-2 (**a**) and loop-1 (**b**) in 7O2K (model 1) and the hexagonal crystal form of CT13Nn (subunit B). 7O2K is colored blue, the subunits A and B of CT13Nn are colored magenta and cyan, respectively. The subunits B and C of CT13Nn are colored orange (**a**) and green (**b**), respectively.

Comparison of subunit E with the NMR structure of CT2Nk (in aqueous solution) showed that the conformations of the loops were very similar (Figure 3a,b). Unusual conformation of the loop-2 in all subunits of the hexagonal and most subunits of the orthorhombic form was similar to that found by NMR for cytotoxin 1 from *N. oxiana* (5NQ4) in DPC micelle [22]. Superimposition of the models (except E) over residues 22–36 resulted in RMSD values in the range of 0.8 to 1.1 Å. Specifically, for model E the respective RMSD value was 1.8 Å. Superimposition of subunit E and A of CT13Nn demonstrated that the tip of the loop-2 was repulsed from the hydrophobic amino acid residues 8–9 of loop-1 of the neighboring subunit B due to crystal packing (Figure 2). We found that the tightly bound water molecule was present within the loop-2 of CT13Nn, similarly to that in CT2Nk structure (pdb 7O2K). Due to the resolution limit, this water molecule was found in all subunits of the hexagonal crystal form and only in two subunits of the orthorhombic crystal form. This molecule was H-bonded to the following atoms: N of Met26, O of Val32, and OG of Thr31 (Figure 3a). In contrast, in the NMR structure 7O2K this water molecule formed three hydrogen bonds with the N atom of Met 26 and the carbonyls of Val 32 and Asn 29 residues (Figure 3a).

Apparently, the difference in this coordination of the water molecule is caused by the crystal packing effects. In the crystal structure of CT13Nn, Thr31 residue cannot be turned toward solvent (as in the NMR structure) due to proximity of the residues of the symmetrically related molecule (Figure 3a,b). Comparison of the structure of CT2Nk and the crystal structure of S-type toxins (pdb codes: 4OM4, 4OM5, and 1UG4) and P-type toxins (pdb code1H0J) supports this conclusion.

### 2.2. MD Study of CT13Nn and CT1No/HMMM POPG Interactions

Study of the interaction of polycationic peptides with anionic lipid membranes, using conventional force fields is highly time-demanding. To accelerate simulations, we used the Highly Mimetic Membrane Model [38] and considered anionic POPG membrane. To compare effects of HMMM membranes on the spatial organization of CTs, we studied, as a reference molecule, CT1No (Table 1). Thus, CT13Nn (CT2Nk) and CT1No were studied side-by-side in POPG bilayers.

**Figure 4:**
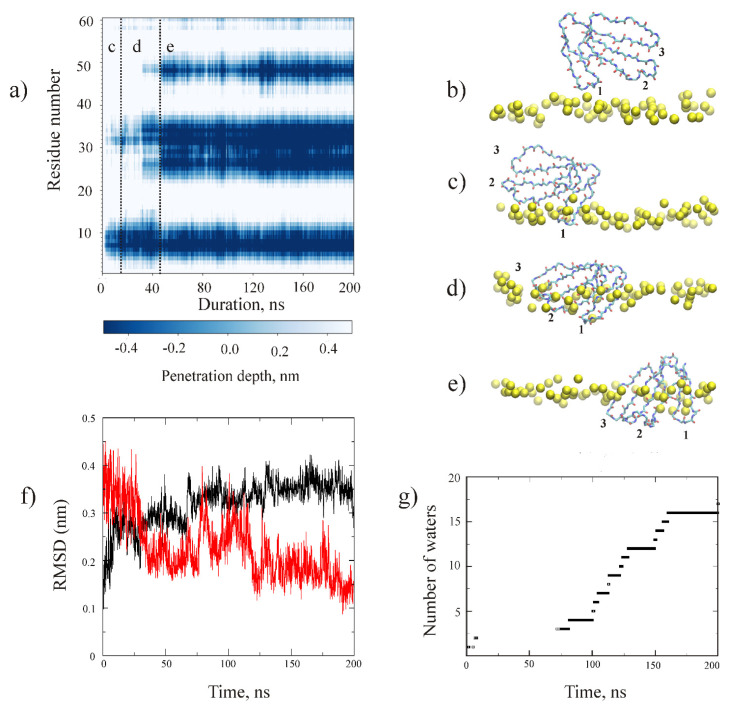
Interaction of CT2Nk (CT13Nn) with the HMMM POPG bilayer. The map of penetration depth of Cα atoms of CT2Nk (**a**) and modes of its interaction with the membrane (**b**–**e**). The penetration depth of the Cα atoms of CT2Nk relative to the average position of PO4- moieties of the phospholipid molecules is shown in color, according to the scale given below the panel (**a**). Snapshots of the CT2Nk/HMMM POPG system during simulation (**b**–**e**). Only backbone atoms of CT2Nk and phosphate atoms of POPG molecules from the upper leaflet, shown in yellow spheres, are drawn. Panel b corresponds to the starting position of the CT2Nk molecule relative to the membrane (zero time in MD). Panels (**c**–**e**) correspond to the respective regions (marked with letters c–e) on the map in the panel (**a**). The loops of CT2Nk are numbered consecutively from 1 to 3 in the panels (**b**–**e**). The numbers situated below the phosphate atoms of the membrane leaflet correspond to the respective loop, embedded in the bilayer. No DCLE molecules are shown. (**f**) Interaction of CT2Nk with the HMMM POPG membrane. Variation of the backbone of residues 22–36 of the toxin during its interaction with the HMMM POPG bilayer (**f**). The black curve is calculated respective to the solution conformation of the toxin molecule (pdb code 7O2K). The red curve is calculated respective to the micelle-embedded conformation of CT1No (5NQ4). (**g**) Exchange of water molecules in the cavity of the loop-2 of CT2Nk in the course of the MD simulation. The water molecules form either two or three hydrogen bonds to the following atoms: the HN group of Met26, the CO group of Val32, and the side-chain OH group of Thr31, or the HN group of Met26 or the CO group of Val32.

First, we simulated the interaction of CT2Nk (model 7O2K) with the HMMM POPG bilayer (Figure 4).

**Figure 5:**
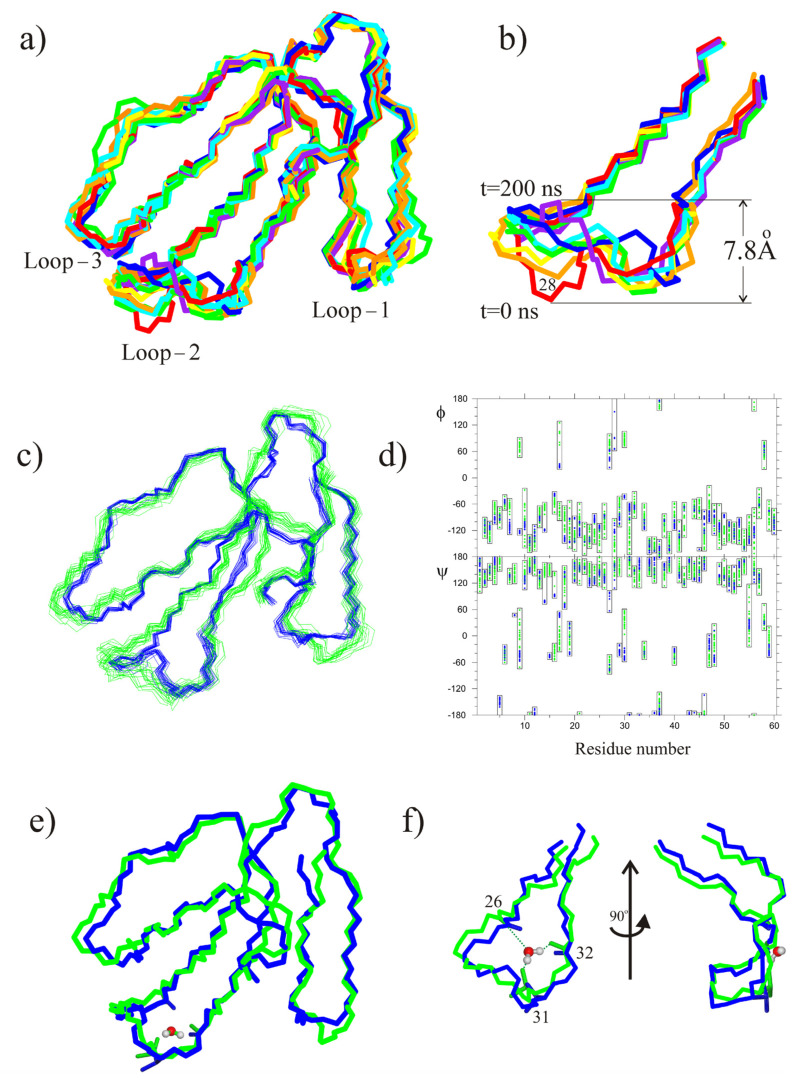
Variation of the 3D-structure of CT2Nk in the course of MD simulation in the presence of the HMMM POPG bilayer and its comparison with the spatial structure of CT1No in the detergent micelle. (**a**) The toxin conformations (superimposed over the backbone atoms of residues 1–60) extracted from the MD trajectory are shown at time = 0 ns (red), 12 ns (orange), 20 ns (yellow), 38 ns (green), 56 ns (cyan), 70 ns (blue), and 200 ns (dark violet) (**a**). Loops 1–3 are marked. See Figure 2, a for attribution of the loops in the amino acid sequence of the toxin. (**b**) The residues 22–36 of the structural ensemble from the panel (**a**) are shown in panel (**b**). Time marks corresponding to the red model and dark violet one are shown. The distance scale is shown on the right. The position of the Ser28 residue is marked. Comparison of the CT2Nk structure from the MD simulation in the HMMM POPG membrane (20 models, green color) and one of the CT1No in DPC micelles (pdb code 5NQ4, 20 models, blue color). (**d**) Comparison of PHI and PSI backbone angles of the ensembles shown in the panel (**c**). The color coding is the same in the both panels. For each residue, the interval of the angle variation is enclosed in the boxes. (**e**) A tightly bound water molecule in the cavity of the loop-2 is shown. (**f**) Two different views of the residue fragment 22–36 are shown in the panel (**e**). Two side views obtained by the rotation around the vertical axis are shown on the panel (**f**). Hydrogen bonds between the backbone and side-chain atoms of the toxin molecule and tightly-bound water molecule within the cavity of this loop are indicated with dotted lines. The number 26 corresponds to the NH bond of the 26th residue, 32 with the CO group of the 32nd residue, and 31 with the side-chain OH group of the 31st residue.

The toxin molecule was placed outside the bilayer, as shown in Figure 4b. The time dependence of the deepening of Cα atoms of the toxin molecule is shown in Figure 4a. As can be seen from this map, the toxin molecule interacted with the bilayer, inserting consecutively loop-1, then loop-2, and finally, loop-3 (Figure 4c–e). Interestingly, insertion of loop-2 was accompanied by structural changes (Figure 5).

The time dependence of these changes (Figure 4f and Figure 5a,b) suggests that they start when the toxin molecule embeds its loop-2 into the membrane, i.e., in the beginning of the period marked with a letter “d” on Figure 4a. These structural changes leave intact the cavity within loop-2 for the tightly-bound water molecule (Figure 4g).

We note that the final conformation of loop-2 in the HMMM POPG membrane is very similar to that of the respective fragment of CT1No in DPC micelle (Figure 5c,d). Thus, the question arises as to whether interaction of CT1No with the HMMM POPG bilayer would result in structural transformations, similar to those observed during its embedding in DPC micelle. This has recently been described [22]. To answer this question, we performed MD simulation of CT1No in the presence of the HMMM POPG bilayer. The starting conformation of the molecule was one of the major forms of CT1No determined by NMR in aqueous solution (pdb code 1RL5, Table 3). Its position and orientation toward the bilayer were identical to that of CT2Nk (Figure 4b). In this case, we obtained the map of penetration, which was nearly identical to that observed for CT2Nk (Figure S3a). Within ~50 ns CT1No embedded all its three loops into the membrane. Close inspection of the MD trajectory revealed that the insertion was accompanied by structural changes, which were more pronounced in the loop-2 region, as in the case of CT13Nn (Figure 4f). The equilibrium conformation of this loop was similar to that obtained in DPC micelle (pdb code 5NQ4) (Figure S4). Thus, embedding of CT1No in the HMMM POPG bilayer resulted in structural changes, similar to those observed by NMR spectroscopy for this toxin in the detergent micelle. Variation of the loop-2 of CT1No (Figure S4) was similar to that shown for CT2Nk in Figure 4f.

In addition, we performed MD simulations of either CT1No or CT2Nk in the presence of the HMMM POPG bilayer, starting from the micelle-embedded conformation for the former toxin (pdb code 5NQ4), or one adapted to the HMMM POPG bilayer for the latter. We found that the molecule did not change its conformation in the course of MD simulation. Embedding of the loops 2 and 3 occurred quickly and simultaneously. This contrasts with the situation when simulation was started with the “water” conformation, where loop-3 embedded after loop-2 with a delay (Figure 4a and Figure S3). Interestingly, the hydrogen-bonding pattern of the tightly-bound water molecule did not change in the course of the MD run (Figure S5). Thus, membrane adaptation of the loop-2 conformation accelerated reaching of the binding mode with the all three loops of CTs embedded into the membrane.

Taking the data for CT1No into account, we may suppose that relatively fast embedding of CTs into the HMMM POPG bilayer is accompanied with structural changes within the loop-2 region, similar to those observed in embedding of this molecule in detergent micelle.

## 3. Discussion

Among the spatial structures of CTs the quality of the NMR-determined models is usually inferior to that of X-ray ones [7]. One of the reasons for this is slow conformational equilibrium between cis- and trans-isomers of the X-Pro8 peptide bond, which occurs in aqueous solution but not in the crystal [39,40]. This equilibrium results in an increase in the number of cross-peaks in NMR spectra of the toxins in aqueous solution. Normal mode analysis [7], MD study [39], and NMR investigation [41] of the dynamics of CTs revealed that the residues involved in beta-sheet formation, are more rigid compared to those that are not involved in backbone hydrogen bonding. The latter are localized in the tips of loops (residues 7–11, 16–18, 28–31, and 46–48). Three of these moieties are involved in binding to lipid membranes: loop-1 (residues 7–11), loop-2 (residues 28–31), and loop-3 (residues 46–48) [22,26,42]. The conformation of these fragments differs in aqueous and membrane environments [21,22]. However, the most significant changes are observed in the tip of loop-2 [22]. This fragment adopts a configuration of an omega loop, a widespread structural motif of the globular protein’ secondary structure [43,44].

For the first time, in this work the structure similar to the membrane-bound conformation of functionally important loop-2 of an S-type CT was observed in the two crystal forms. Apparently, these conformational changes are caused by the repulsion of loop-2 from its neighbor molecules in the crystal cell. One can assume that the hydrophobic residues of the neighbor “mimic” in some way the membrane surface and sterically disturb the tip of loop-2. Earlier, related effects were observed and interpreted in a similar way in the X-ray structure of cardiotoxin-like basic protein A5 from Taiwan cobra venom [45]. Polymorphism of protein crystals is not a rare event. Polymorphic crystals formed under similar conditions make it possible to identify crystal structures with potentially similar free energies of crystallization [46].

Despite some amino acid variability of the loop-2 in different CTs, only two conformations (aqueous and membrane-bound) could be found. This indicates the structural role of the conservative tightly bound water molecule found in NMR and X-ray structures. Similarity coordination of this water molecule within the hexagonal X-ray structure and NMR-structure of CT1 *N. oxiana* in the presence of DPC (5NQ4) is in a good agreement with this suggestion. In the structure 5NQ4, the side chain of Thr 31 is turned toward this water molecule in a manner similar to our crystal structure. Therefore, the H bond of this residue with tightly bound water in the “membrane” form cannot be excluded. Of note, the amino acid sequences of the loop-2 of these toxins differed slightly. This resulted in minor differences in the loop conformation and coordination of the water molecule within it (Figure S2).

In this work, we used the HMMM model to investigate the interaction of CT2Nk with POPG membrane. For comparison, we studied the interaction of CT1No with the membrane, because the spatial structures of this toxin have been determined with NMR spectroscopy both in aqueous solution and in detergent micelles (Table 2). We found that interaction of the both CT2Nk and CT1No toxins with this membrane proceeds via similar steps, detected in AA-study of cytotoxin 2 from *N. oxiana* in POPC membrane [31]. Firstly, all these toxins insert in the membranes loop-1, then loop-2, and finally, loop-3, in accordance with hydrophobicity gradient between these loops [47]. In POPG membrane, incorporation of loop-2 was accompanied by its structural remodeling. Interestingly, the final conformational state of loop-2 in POPG membrane was similar to the corresponding fragment of CT1No in DPC micelle (Figure S3b). When the starting conformation of CT1No or CT2Nk was one in either DPC micelle or HMMM POPG membrane, no changes of the loop-2 occurred after embedding of the toxin molecule in the HMMM POPG bilayer (Figure S3b). Also, in this case the hydrogen bonding pattern of the tightly bound water molecule within loop-2 did not change in the course of the MD simulation (Figure S5). This is clearly different to the case of embedding of CT2Nk in the HMMM POPG membrane starting from its solution conformation (Figure 4a). The hydrogen-bonding pattern of a tightly bound water molecule in the loop-2 changed (Figure 4g) because the tip of loop-2 reorganized in the membrane (Figure 5a,b). Both CT1No and CT2Nk are S-type CTs [20]. Thus, the membrane effect on the loop-2 of CTs can be reproduced in silico using HMMM approximation of anionic membranes and CHARMM36m force field. This force field has been shown to correctly reproduce protein–lipid interactions at the membrane interface [48]. At the same time, this force field introduces some flexibility in the membrane-interacting loops of CTs. Indeed, the dihedral angle PHI of some residues from loop-1 and loop-2 of CT2Nk in the HMMM POPG bilayer (Figure 5d) took positive values, unlike those in the aqueous phase (Figure 5d). However, this flexibility seems to be important to correctly reproduce adaptation of the CT loops to the membrane environment.

The question arises, as to whether the membrane adaptation of the loop-2 depends on the lipid composition of the membrane. Our previous analysis showed that the modes of interaction of CTs with zwitterionic and anionic lipid membranes are similar [27]. Previous study of the interaction of P- and S-type CTs with liposomes, formed of PG lipids showed that classification into P- and S-types can be expanded from zwitterionic to anionic lipid membranes [27]. Namely, the binding modes of these toxins to either zwitterionic, or anionic membranes do not differ. In the both cases the three-loop mode is realized, although the membrane binding constants differ. Most probably, the molecular basis for that is caused by the fact that the tip of the loop-2 of S-type CTs is formed of more hydrophilic residues, compared to those of their P-type counterparts [20]. As a result, the conformation of the tip of this loop of S-type CTs changes in the membrane, while in P-type ones it remains intact upon incorporation into micelle [21], or lipid membrane [31]. As we see in the current work, changes of the conformation of the loop-2 in S-type CT1No observed upon their transfer from aqueous solution to detergent micelle, or HMMM POPG membrane are very similar. Thus, simulation of such toxins with these membrane models can substitute in many aspects their study by NMR spectroscopy in detergent micelles.

There are numerous reports on studies of cytotoxic activity of cobra CTs, including their action on cancer cell lines (e.g., [49,50]). For some of the latter, P-type CTs possess higher activity, compared to their S-type counterparts [19]. In this case we may assume that this is due to different efficiency of the interaction of these CTs with either the plasma membrane, or with membranes of intracellular organelles of the cancer cells. S-type CTs require an additional step during their interaction with membranes. Our data indicate that in the process of membrane-binding, the structure of an S-type CT molecule undergoes rearrangement, or adaptation to the membrane environment. The P-type CTs do not require rearrangement of their loop-2 [21,31]. We believe that in cases where the membrane activity of CTs underlies their cytotoxic mechanism, the P-type CTs possess higher activity, compared to S-type ones. This rule will remain true in considering toxicity of CTs in animal tissues. Recently, it was demonstrated that CTs of either P- or S-types influenced the function of blood vessel and heart muscle [51]. This finding again indicates that the membrane-affected structure of CTs is an important factor in their toxic effects. We believe that the observed conformation of CT loops in the membrane-bound molecule should be taken into account in the design of cytotoxic drugs based on CTs. At the same time, we cannot exclude that not only cell membranes but also some membrane proteins are affected by CTs. In this case, both hydrophobic and electrostatic properties of CT molecules should be accurately taken into account [49,52].

## 4. Conclusions

For the first time, in the crystal state of a CT molecule, the membrane-bound conformation of its central loop has been observed. Polymorphism of protein crystals together with MD studies and analysis of NMR structures of homologues assists in elucidating functional features of CTs. The HMMM bilayer can be used as a membrane environment, where adaptation of peripheral polypeptides to the lipid–water interface can be studied in atomistic detail via molecular dynamics simulations.

## 5. Materials and Methods

### 5.1. Cytotoxin Purification

CT13Nn was isolated from *N. naja* venom and purified essentially as described in [53]. The purification was performed in three steps, including gel-filtration, ion-exchange and reverse-phase high-performance liquid chromatography. Briefly, gel filtration was performed on a Superdex 75 column (10 × 300 mm, Cytiva, Marlborough, MA, USA), equilibrated with 0.1 M ammonium acetate (pH 6.2), at a flow rate of 0.5 mL/min. The main toxic fraction containing cytotoxins was separated by ion-exchange chromatography on a HEMA BIO 1000 CM column (8 × 250 mm, Tessek, Prague, Czech Republic) with a gradient of 5–700 mM ammonium acetate (pH 7.5) for 140 min, at a flow rate of 0.5 mL/min. Fractions containing cytotoxins were further separated by reversed-phase chromatography on a Bio Wide Pore C18 column (10 × 250 mm, Merck KGaA, Darmstadt, Germany) in a 20–50% gradient of acetonitrile for 60 min in the presence of 0.1% trifluoroacetic acid, at a flow rate of 2.0 mL/min. The amino acid sequence of the isolated toxin was analyzed by mass spectrometry, as described [54].

### 5.2. Crystallization, Data Collection, and Processing

The initial conditions for CT13Nn (5 mg/mL) crystallization were screened at room temperature by the vapor-diffusion method using screening kits Crystal Screen HR2–110 and Crystal Screen HR2-112 (Hampton Research). The final optimized crystals were grown by vapor-diffusion or counter-diffusion methods from (1.7–1.9 M sodium chloride, 0.1 M sodium dihydrogen phosphate, 0.1*M* MES, pH 5.5).

For data collection, the crystals were soaked in a cryoprotectant solution consisting of reservoir solution with 20% glycerol added. The crystals were then flash-cooled in liquid nitrogen at 100 K. Diffraction data for the orthorhombic crystal form were collected at beamline P14, Deutsches Elektronen-Synchrotron (DESY), Petra III, Hamburg, Germany, and for the hexagonal crystal form at beamline BL41XU at SPring-8, Japan. Data were processed by XDS [55] or HKL2000 [56]. Data collection statistics, along with unit cell dimensions, are summarized in Table 2.

### 5.3. Structure Determination and Refinement

Structures of hexagonal and orthorhombic crystal forms were solved at 2.3 and 2.6 Å, respectively. The structure was determined by molecular replacement with Phaser [57] using the structure of the CT1No (PDB code 1RL5) as the initial model. Hexagonal and orthorhombic forms contain three or six subunits in the asymmetric part, respectively. Model refinements were performed with Refmac5 [58], combined with manual model-rebuilding using Coot [59]. The models of hexagonal and orthorhombic crystal forms were refined to a crystallographic R-factor of 19.7% (R_free_ = 24.2%) and R-factor of 20.49% (R_free_ = 24.8%), respectively. Model validation was performed with PROCHECK [60]. Refinement statistics are summarized in Table 2. Structures were deposited with the Protein Data Bank under ID of 7QHI and 7QFC for hexagonal and orthorhombic forms, respectively.

### 5.4. Molecular Dynamics

The cytotoxin/membrane systems were assembled with the use of CHARMM-GUI Membrane Builder, or input generator (http://charmm-gui.org/?doc=input/membrane.bilayer, accessed on 16 February 2022) [61]. The CT molecule was placed outside the bilayer composed of 128 lipid molecules in an orientation in which the tip of loop-1 was directed toward the membrane surface (see further details). The starting conformation of the toxins was determined by either X-ray or NMR methods, or derived from MD simulations (Table 3). To check reproducibility of the simulations, three independent runs were performed for each toxin/lipid system listed in Table 3. In these starts, the orientations of the toxin molecules with respect the membrane surface were identical, but the distance from the center of mass of the toxin molecule to the center of the lipid bilayer was varied in the range 37–45 Å.

The parameters of the CT13Nn (CT1No)/lipid assemblies are given in the Supplementary Materials (Table S2). The per-lipid area ratio (R_SA_, ratio of the average area per lipid molecule to that in the all-atom model of the membrane) was chosen to be a default value of 1.1. The terminal acyl carbon number was fixed at the 6th carbon atom. This means that the lipid molecules were truncated below the 6th carbon atom and the free membrane volume was replaced with a box of organic solvent, 1,1-dichloroethane (DCLE). In all calculations, the tip3p water model was employed [62]. To keep the system electrically neutral, counterions were added (see Table S3). The CHARMM36m force field was used to perform MD simulations. The following simulation parameters were selected: the NPT ensemble, i.e., a constant number of molecules in the simulation box, pressure and temperature. Van der Waals interactions were smoothly switched off at 10–12 Å by a force-based switching function [63]. The particle-mesh Ewald algorithm was used to evaluate electrostatic interactions [64]. A time step of 2 fs was used in all simulations. The temperature was kept at 303.15 K using a Nosé–Hoover thermostat. The Nosé–Hoover Langevin-piston method [65,66] was used to maintain constant pressure at 1 bar. The standard CHARMM-GUI six-step protocol was used to equilibrate the systems. The length of MD trajectories was 200 ns. Other details of MD simulations and data analysis were similar to those used previously [67].

## Figures and Tables

**Table 1:** Amino acid sequences of CTs studied in this work.

Toxin Name/UNIPROT Code	Amino Acid Sequence (with Numbering above the Sequence)
	1 5 10 15 20 25 30 35 40 45 50 55 60
CT3Nk ^1^/P01446	LKCNKLIPLAYKTCPAGKNLCYKMFMVSNKTVPVKRGCIDACPKNSLLVKYVCCNTDRCN
CT13Nn ^2^	LKCNKLIPLAYKTCPAGKNLCYKMFMVSNKTVPVKRGCIDVCPKNSLLVKYVCCNTDRCN
CT1No ^3^/P01451	LKCNKLVPIAYKTCPEGKNLCYKMFMMSDLTIPVKRGCIDVCPKNSLLVKYVCCNTDRCN
CT3Nn/P24780	LKCNKLIPLAYKTCPAGKNLCYKMFMVSNKTVPVKRGCIDVCPKNSLVLKYVCCNTDRCN

^1^ cytotoxin 3 from *N. kaouthia*; ^2^ mature cytotoxin 13 from *N. naja* (A0A0U4N5W4), identical to cytotoxin 2 from *N. kaouthia* CT2Nk (P01445) and named in this work as L48V49 CT3Nn; ^3^ cytotoxin 1 from *N. oxiana*; residues that are different from those in CT3Nk are underlined.

**Table 2:** Data collection and refinement statistics.

Crystal Form	Hexagonal	Orthorhombic
Data collection		
Synchrotron	SPring-8	DESY, Petra III
Wavelength (Å)	1.000	0.9763
Space group	P6_4_22	C222_1_
*a*, *b*, *c* (Å)	109.33, 109.33, 135.77	88.74, 97.17, 132.12
α, β, γ (°)	90, 90, 120	90, 90, 90
Resolution range (Å)	50.0–2.30 (2.36–2.30) *	50.0–2.50 (2.63–2.60) *
*R*_mege_ (%)	10.4 (54.7)	18.1 (89.4)
<*I*/σ(*I*)>	13.4 (2.3)	10.7 (1.8)
Completeness (%)	98.39 (89.48)	99.17 (99.93)
Redundancy	8.2 (7.2)	6.3 (3.4)
**Refinement**		
Resolution (Å)	14.95–2.30 (2.36–2.30) *	14.91–2.60 (2.66–2.60) *
Number of reflections	20,428	18,995
R_work_/R_free_ (%)	19.7/24.2	20.5/25.5
No. atoms		
Protein	1392	2784
Water	218	17
B-factors		
Protein	38.11	98.51
Solvent	35.98	43.93
RMSD		
Bond lengths (Å)	0.013	0.013
Bond angles (°)	2.094	2.191
Ramachandran statistics		
Most-favored regions (%)	94.3	89.4
Allowed regions (%)	4.0	8.3
Disallowed regions (%)	1.7	2.3

* Values in parentheses are for the highest-resolution shell.

**Table 3:** Starting conformations of CTs used in MD simulations.

Toxin			
CT1No ^a^		CT13Nn ^a^	
Solution ^b^	Membrane ^c^	Solution ^b^	Membrane
1RL5	5NQ4	7O2K	7QHI (model A)

^a^ see Table 1 for the amino acid sequences of the toxins; ^b^ solution conformations of the major form of CT1No [22,39] or CT2Nk [37]; and ^c^ conformation of CT1No in detergent micelles [22].

## Data Availability

X-ray structures of cytotoxin 13 from *N. naja* have been deposited with the Protein Data Bank (codes 7QFC, 7QHI).

## Supplementary Materials

The following supporting information can be downloaded at: https://www.mdpi.com/article/10.3390/toxins14020149/s1, Table S1: Overall B-factors of protein chains of L48V49 CT3Nn the crystal forms, Table S2: Typical parameters of the simulation box and system size in the CT/HMMM POPG systems, Figure S1: The 2Fo-Fc density map at 1.2σ level of 47–52 fragment of subunit A of 7QFC, Figure S2: Conservative water molecule in the loop-2 of cobra toxins. Superposition of subunit B of XXX3(cyan) and 5NQ4(pink). Water molecule is shown by red sphere in 7QFC and dark red sphere in 5NQ4, Figure S3: Interaction of CT1No in different starting conformations with the HMMM POPG bilayer. (a) Solution conformation, pdb code 1RL5, (b) conformation adopted in detergent micelle, 5NQ4. The model #1 from the respective NMR ensem-bles are shown to the right of the panels (blue color). Superimposed by backbone atoms of 1–60 residues are shown, in addition, solution conformation of CT2Nk (pdb code 7O2K) (panel a) and equilibrium conformation of CT2Nk molecule in the course of MD simulation in HMMM POPG bilayer (see Figure 4, panel a for details). The CT2Nk molecules are shown in red color. The loops are marked in the upper panel. The orientation of the superimposed models is the same in the both panels. The penetration depth of the CA-atoms of CT1No relative to the average position of PO4- moieties of the POPG molecules is shown in color, according to the scale given below the panel (b). Note that the loop-3 of the toxin molecule embeds in the bilayer simultaneously with the loop-2 in the panel (b), while in the panel (a) the loop-3 embeds the bilayer with a delay respective to the loop-2, Figure S4: Interaction of CT1No with POPG bilayer probed by time-dependence of the variation of backbone RMSD for residues 22-36. The starting MD-state of the molecule corresponds to its “water” conformation (1RL5) and the black curve is calculated relative to it. The red curve is calculated relative to the “membrane” conformation of the molecule (5NQ4). First, the molecules from trajectory and the template were superimposed over all backbone atoms. Then the RMSD over 22-36 residues was calculated, Figure S5: Exchange of water molecules in the cavity of the loop-2 of CT1No in the course of the MD simulation in HMMM POPG bilayer. The starting conformation of the molecule was one determined by NMR in detergent micelle (pdb code 5NQ4). The respective deepening map of CA atoms is shown in Figure S3b. The horizontal bars correspond to the hydrogen bonding to acceptor backbone atoms, indicated in the insert in the top upper part of the graph. Note that these hydro-gen bonds exist practically during the whole duration of the trajectory, unlike for CT2Nk started from its solution con-formation (Figure 4g).

## Abbreviations

2D, two-dimensional; 3D, three-dimensional; AA, all-atom; CT(s), cytotoxin(s); CT1No, CT1 from *N. oxiana*; CT13Nn, CT13 from *N. naja*; CT2Nk, CT2 from *N. kaouthia*; CT3Nk, CT3 from *N. kaouthia*; CG, coarse-grained; DCLE, 1,1-dichloroethane; DPC, dodecylphosphocholine; HMMM, Highly Mimetic Membrane Model; MD, molecular dynamics; POPC, palmitoiloleoylphosphatidylcholine; POPG, palmitoiloleoylphosphatidylglycerol; RMSD, root-mean-square deviation; TFT, three-finger toxin.
