# Supplementary material for: Variability in the Spatial Structure of the Central Loop in Cobra Cytotoxins Revealed by X-ray Analysis and Molecular Modeling
Source: Toxins (Basel). 2022 Feb 18;14(2):149. doi: 10.3390/toxins14020149 (PMC8880459; doi:10.3390/toxins14020149)
Supplement: Supplementary file 1 [file toxins-14-00149-s001.zip › toxins-1555038-supplementary.pdf]

Supplementary Materials:

Variability in the Spatial Structure of the Central Loop in Cobra Cytotoxins Revealed by X-Ray Analysis and Molecular Modeling

Peter V. Dubovskii, Kira M. Dubova, Gleb Bourenkov, Vladislav G. Starkov, Anastasia G. Konshina, Roman G. Efremov, Yuri N. Utkin and Valeriya R. Samygina

Table S1. Overall B-bactors of protein chains of L48V49 CT3Nn crystal forms.

B-factor, Å^2	Hexagonal crystal form	Orthorhombic crystal form
Subunit A		74.323
Subunit B	34.50	80.634
Subunit C	31.08	82.392
Subunit D	49.44	102.39
Subunit E		121.30
Subunit F		130.06

Table S2. Typical parameters of the simulation box and system size in the CT/HMMM POPG systems.

Cell size (x-y-z, nm)	Number of lipid molecules (upper/lower leafleats)	Number of water molecules[a]	Number of ions[b]	Total number of atoms/ number of DCLE[c] molecules
7.37x7.37x8.87[d]	64/64	9842	119	43835/647
7.37x7.37x9.24[e]	64/64	10682	119	46355/647

[a] In all calculations the TIP3P water model was used; [b] Na+ ions, no any salt was added;[c] DCLE, 1,1-dichloroethane;[d] The distance from the center mass of the toxin molecule to the center of the bilayer, D= 37 Å, orientation of the toxin molecule is shown in Fig.4, b; [e] D=43Å.

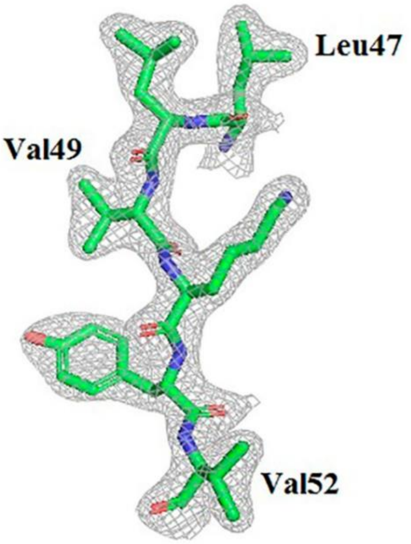

Figure S1. The 2Fo-Fc density map at 1.2σ level of 47-52 fragment of subunit A of 7QFC.

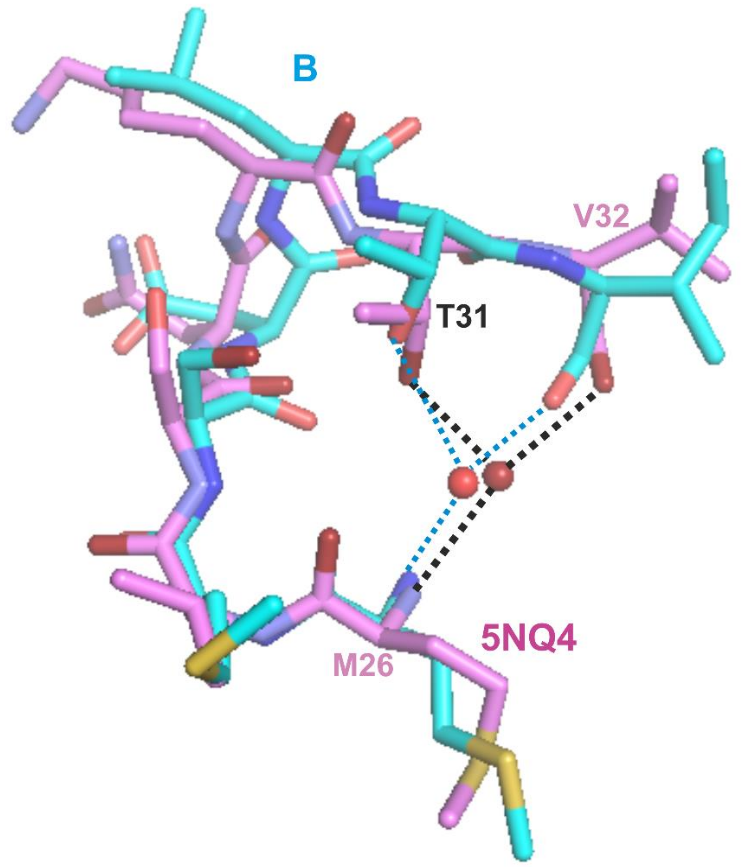

Figure S2. Conservative water molecule in the loop-2 of cobra toxins. Superposition of subunit B of XXX3(cyan) and 5NQ4(pink). Water molecule is shown by red sphere in 7QFC and dark red sphere in 5NQ4.

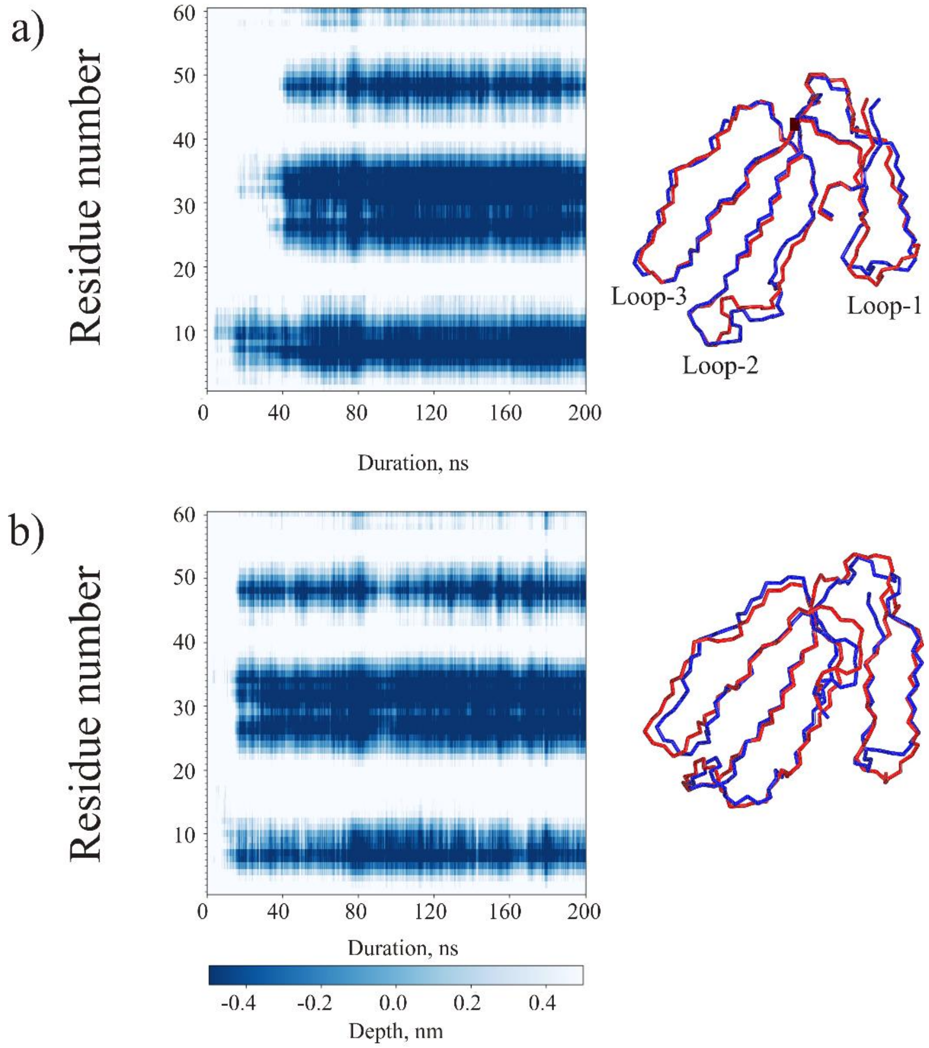

Figure S3. Interaction of CT1No in different starting conformations with the HMMM POPG bilayer. (a) Solution conformation, pdb code 1RL5, (b) - conformation adopted in detergent micelle, 5NQ4. The model #1 from the respective NMR ensembles are shown to the right of the panels (blue color). Superimposed by backbone atoms of 1-60 residues are shown, in addition, solution conformation of CT2Nk (pdb code 7O2K) (panel a) and equilibrium conformation of CT2Nk molecule in the course of MD simulation in HMMM POPG bilayer (see Figure 4, panel a for details). The CT2Nk molecules are shown in red color. The loops are marked in the upper panel. The orientation of the superimposed models is the same in the both panels. The penetration depth of the CA-atoms of CT1No relative to the average position of PO4- moieties of the POPG molecules is shown in color, according to the scale given below the panel (b). Note that the loop-3 of the toxin molecule embeds in the bilayer simultaneously with the loop-2 in the panel (b), while in the panel (a) the loop-3 embeds the bilayer with a delay respective to the loop-2.

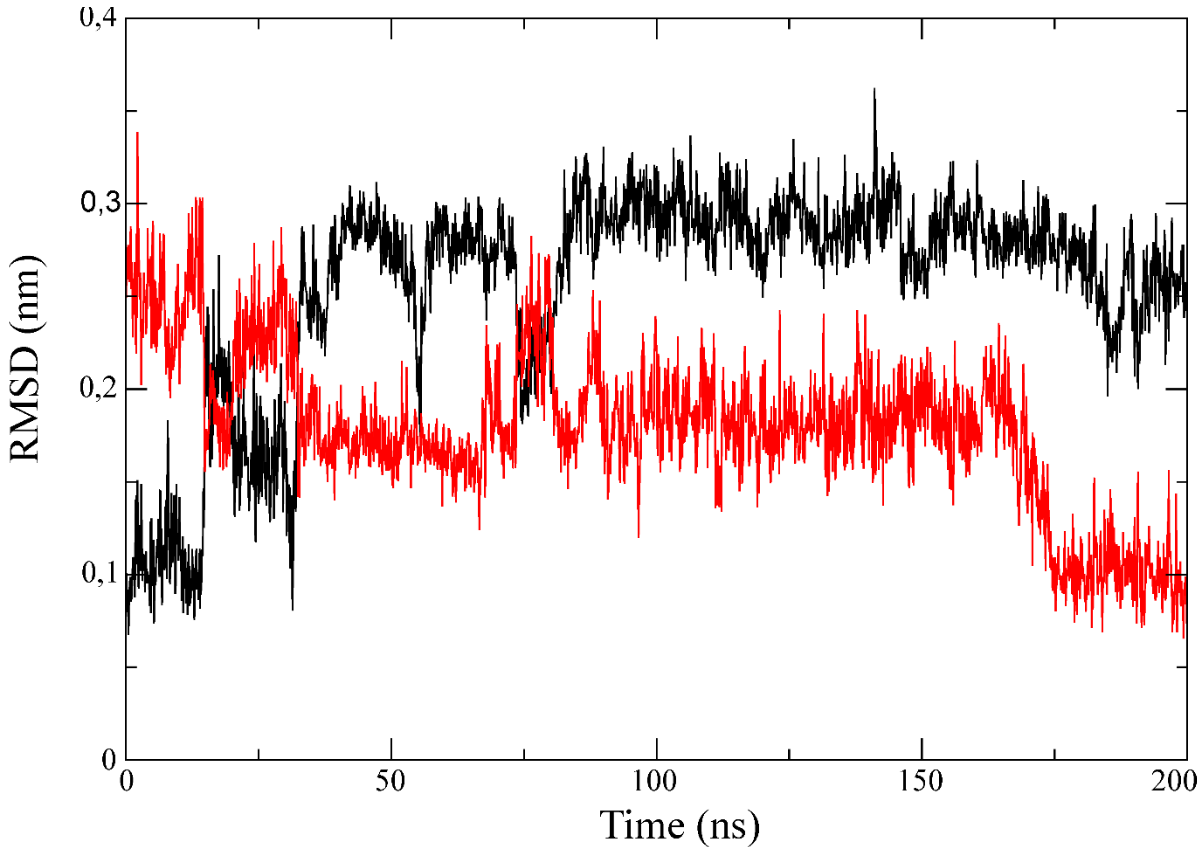

Figure S4. Interaction of CT1No with POPG bilayer probed by time-dependence of the variation of backbone RMSD for residues 22-36. The starting MD-state of the molecule corresponds to its "water" conformation (1RL5) and the black curve is calculated relative to it. The red curve is calculated relative to the "membrane" conformation of the molecule (5NQ4). First, the molecules from trajectory and the template were superimposed over all backbone atoms. Then the RMSD over 22-36 residues was calculated.

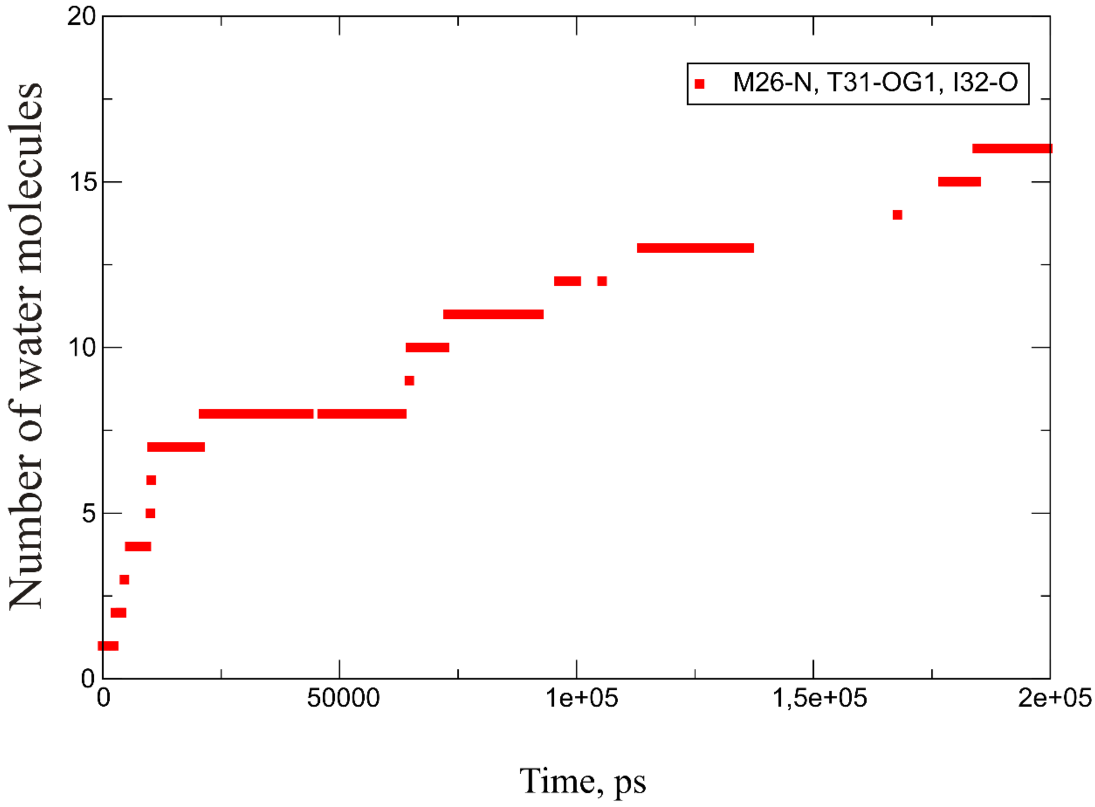

Figure S5. Exchange of water molecules in the cavity of the loop-2 of CT1No in the course of the MD simulation in HMMM POPG bilayer. The starting conformation of the molecule was one determined by NMR in detergent micelle (pdb code 5NQ4). The respective deepening map of CA atoms is shown in Figure S3 b. The horizontal bars correspond to the hydrogen bonding to acceptor backbone atoms, indicated in the insert in the top upper part of the graph. Note that these hydrogen bonds exist practically during the whole duration of the trajectory, unlike for CT2Nk started from its solution conformation (Figure 4 g).
